# Early coronary angioplasty fails to lower all‐cause mortality in patients with out‐of‐hospital cardiac arrest without ST‐segment elevation: A systematic review and meta‐analysis

**DOI:** 10.1002/hsr2.1379

**Published:** 2024-01-30

**Authors:** Khalid Rashid, Muhammad Aamir Waheed, Farrukh Ansar, Abdelrahman M. Makram, Ahmedyar Hasan, Shahab Ahmed, Saad Tariq Khan, Aamer Ubaid, Ahmad Al Ibad, Rabia Basri, Omar Mohamed Makram, Yahya Khan, Nabhan Rashad, Abdelnaser Elzouki

**Affiliations:** ^1^ Internal Medicine James Cook University Hospital Middlesbrough UK; ^2^ Department of Medicine Hamad Medical Corporation Doha Qatar; ^3^ Department of Medicine Quaid e Azam International Hospital Islamabad Pakistan; ^4^ Public health, School of Public Health Imperial College London London UK; ^5^ Department of Anesthesia and Intensive Care Medicine October 6 University Hospital Giza Egypt; ^6^ Department of Medicine Mohammed Bin Rashid University of Medicine and Health Sciences Dubai UAE; ^7^ Medicine King Abdullah Teaching Hospital Mansehra Pakistan; ^8^ Internal Medicine Sunderland Royal Hospital Sunderland UK; ^9^ Internal Medicine University of Missouri Kansas City Kansas City Missouri USA; ^10^ Internal Medicine Bannu Medical College Bannu Pakistan; ^11^ Public health, Faculty of Public Health and Policy London School of Hygiene and Tropical Medicine London UK; ^12^ Medicine, Center for Health & Nature Houston Methodist Hospital Houston Texas USA; ^13^ Department of Cardiology October 6 University Hospital Giza Egypt; ^14^ Mardan Medical Complex Mardan Pakistan; ^15^ Department of Medicine Khyber Teaching Hospital Peshawar Pakistan; ^16^ Department of Medicine, Hamad General Hospital Weill Cornell Medicine Ar‐Rayyan Qatar

**Keywords:** coronary angiography, early vs delayed CAG, out‐of‐hospital cardiac arrest, percutaneous coronary intervention

## Abstract

**Introduction:**

Out‐of‐hospital cardiac arrest (OHCA) is defined as the loss of functional mechanical activity of the heart in association with an absence of systemic circulation, occurring outside of a hospital. Immediate coronary angiography (CAG) with percutaneous coronary intervention is recommended for OHCA with ST‐elevation. We aimed to evaluate the effect of early CAG on mortality and neurological outcomes in OHCA patients without ST‐elevation.

**Methods:**

This meta‐analysis and systemic review was conducted as per principles of Preferred Reporting Items for Systematic Reviews and Meta‐analysis (PRISMA) group. A protocol was registered with the International Prospective Register of Systematic Reviews (PROSPERO, Ref No. = CRD42022327833). A total of 674 studies were retrieved after scanning several databases (PubMed Central, EMBASE, Medline, and Cochrane Central Register of Controlled Trials).

**Results:**

A total of 18 studies were selected for the final analysis, including 6 randomized control trials and 12 observational studies. Statistically, there was no significant difference in primary outcome, i.e., mortality, between early and delayed CAG. In terms of the grade of neurological recovery as a secondary outcome, early and delayed CAG groups also showed no statistically significant difference.

**Conclusion:**

Early CAG has no survival benefits in patients with no ST elevations on ECG after OHCA.

## 1 INTRODUCTION

Out‐of‐hospital cardiac arrest (OHCA) is defined as the loss of functional mechanical activity in association with an absence of systemic circulation, occurring outside of a hospital.^1^ While the incidence and outcome of OHCA vary by country,^2^ it affects more than 500,000 patients worldwide each year.^3^ Despite improvements made within the last two decades, mortality and morbidity remain high, with only 1 in 10 surviving to hospital discharge.^4^ With such high mortality rates and poor neurological outcomes,^5^ OHCA remains a major public health burden that requires immediate identification and appropriate management.

The current American and European guidelines recommend immediate coronary angiography (CAG) with percutaneous coronary intervention (PCI) in patients with OHCA who present with ST‐segment elevation.^6^, ^7^ However, in patients with no ST elevation, the role of immediate PCI remains unclear. In the recent TOMAHAWK trial, 554 patients with OHCA without ST‐elevation were randomly assigned to either immediate CAG or delayed CAG. No benefit was demonstrated over immediate angiography compared to the delayed strategy with respect to the 30‐day risk of death from any cause.^8^ Having said that, other studies have found a benefit in early revascularization,^9^, ^10^, ^11^ indicating mixed evidence. Moreover, a correspondence by field experts highlighted that those trials investigating this issue had stringent selection criteria, limiting the external validity of their results.^12^ Therefore, collecting and analyzing the data from the comparative literature, including observational studies with less strict inclusion criteria, can provide more clarity as to what the appropriate intervention should be.

Therefore, in this systematic review and meta‐analysis, we aimed at gathering all the evidence from the available literature to assess the benefit of early revascularization in patients with OHCA without ST elevation. Providing reliable and discrete evidence on this subject matter can help formulate guidelines to improve the outcomes of OHCA.

## 2 MATERIALS AND METHODS

### 2.1 Protocol registration

Our team conducted this meta‐analysis and systemic review as per the principles of the Preferred Reporting Items for Systematic Reviews and Meta‐analysis (PRISMA) statement and the checklist (Table S1). Initially, a protocol was formulated and registered with the International Prospective Register of Systematic Reviews (PROSPERO CRD42022327833) before starting this study.

### 2.2 Search strategy

An extensive search of PubMed, Web of Science, EMBASE, and Cochrane Central Register of Controlled Trials was conducted by a team of two authors (FA, SK). We searched these sources from inception until June 10, 2022. These databases were systemically screened through the construction of a search strategy using the medical subject headings (Mesh). The MeSH terms of “OHCA” OR “Out of hospital cardiac arrest” OR “Out‐of‐hospital cardiac arrest” AND “Percutaneous Coronary Intervention” OR “PCI” OR “Percutaneous Coronary Angioplasty” OR “Percutaneous Coronary revascularization” were used. Our team also reviewed the references and citations of the published studies, meta‐analyses, and systemic reviews to ensure that no relevant literature was left out of the final analysis (manual search).

### 2.3 Eligibility criteria

This study was designed and conducted around the following research question: “Does performing early CAG improve mortality and/or neurological outcomes in patients with OHCA and no ST‐segment elevations on postarrest electrocardiogram (ECG)?”

Therefore, we included randomized control trials (RCTs), observational studies, and cohort studies that reported adults (over 18 years of age) who suffered from cardiac arrest outside of the hospital, diagnosed to have non‐ST segment elevation myocardial infarction (NSTEMI), and with reported mortality and/or neurological outcomes, namely the cerebral performance category scores (CPC), which is a 5‐point standard scale that ranges from good cerebral performance (1) to brain death (5). Letters to the editors, conference abstracts, case reports and case series, pediatric studies, commentaries, and duplicates were excluded from the finalized pool of studies.

### 2.4 Study selection and data extraction

First, the titles and abstracts of the articles obtained from the databases were checked against the eligibility criteria. Then, the full texts of the shortlisted abstracts were retrieved and evaluated. The data from the shortlisted studies was extracted using a standardized form. All these steps were done by two independent reviewers with complete blinding. Then, their work was compared, and a consensus through a formal discussion was reached if differences were detected. In case of disagreements between these authors, a third author (MW) provided input.

The data recorded included the study authors, design, location, population, basic demographics, cardiovascular comorbidities, and nature of cardiac arrest were obtained in addition to data on mortality and neurological outcomes.

### 2.5 Outcomes

The definition of early versus delayed CAG was variable among the included studies and ranged from 2 to 24 h of OHCA. Six studies defined early as performing CAG in less than 2 h.^13^, ^14^, ^15^, ^16^, ^17^, ^18^ One study defined early as less than 3 h.^19^ One study also defined early as less than 6 h^20^ and four studies defined early as less than 24 h.^21^, ^22^ For the purpose of this study, the main analysis was conducted using the studies that defined early versus delayed CAG with cutoff of 2 h. In this analysis, the intervention group was defined as those who underwent early CAG (within 2 h of OHCA) and the control group was defined as those who underwent delayed CAG (more than 2 h of OHCA) or no CAG. Supplementary analyses for studies were conducted for studies that defined early vs delayed CAG with cutoff other than 2 h, with a special focus to the cutoff of 24 h.

The primary outcome of this study was all‐cause mortality, and the secondary outcome was defined as the neurological status of these patients. A CPC score of 1–2 was considered a good neurological outcome. Thirteen studies utilized CPC scoring as the neurological recovery parameter. Crude propensity scoring was used in one study, and in four of the studies there was no available information with regards to neurological status. When CAG indicated a substantial coronary artery lesion, PCI was defined as revascularization with stent insertion.

### 2.6 Statistical analysis

The statistical analysis was carried out using R programming language and the “meta” package.^19^ Data were entered in the format of a comparison between two categories to perform the analysis, and analysis was then carried out for dichotomous data. The categories were named in accordance with the data, which was recorded as the number of incidents in each category. As indicated in the tables, the precise calculations were reported in the odds ratio and 95% confidence interval (CI). A random effect model was employed to perform this meta‐analysis as the analysis included a combination of observational studies and clinical trials. Calculating the Q values, df values, Q‐df values, T, and I^2^ statistics were the techniques utilized to evaluate heterogeneity and variability.

### 2.7 Risk of bias assessment

The risk of bias was assessed using the Cochrane risk‐of‐bias tool for randomized trials (RoB 2)^23^ or the risk of bias in non‐randomized studies of interventions (ROBINS‐I)^24^ in the respective study designs using the same methodology adopted in the study selection and data extraction. Publication bias was neither assessed using Egger's regression test nor visualized using Begg's funnel plot as the number of studies in each forest plot did not exceed 10 studies.^25^, ^26^

## 3 RESULTS

### 3.1 Searching databases

**Figure 1:**
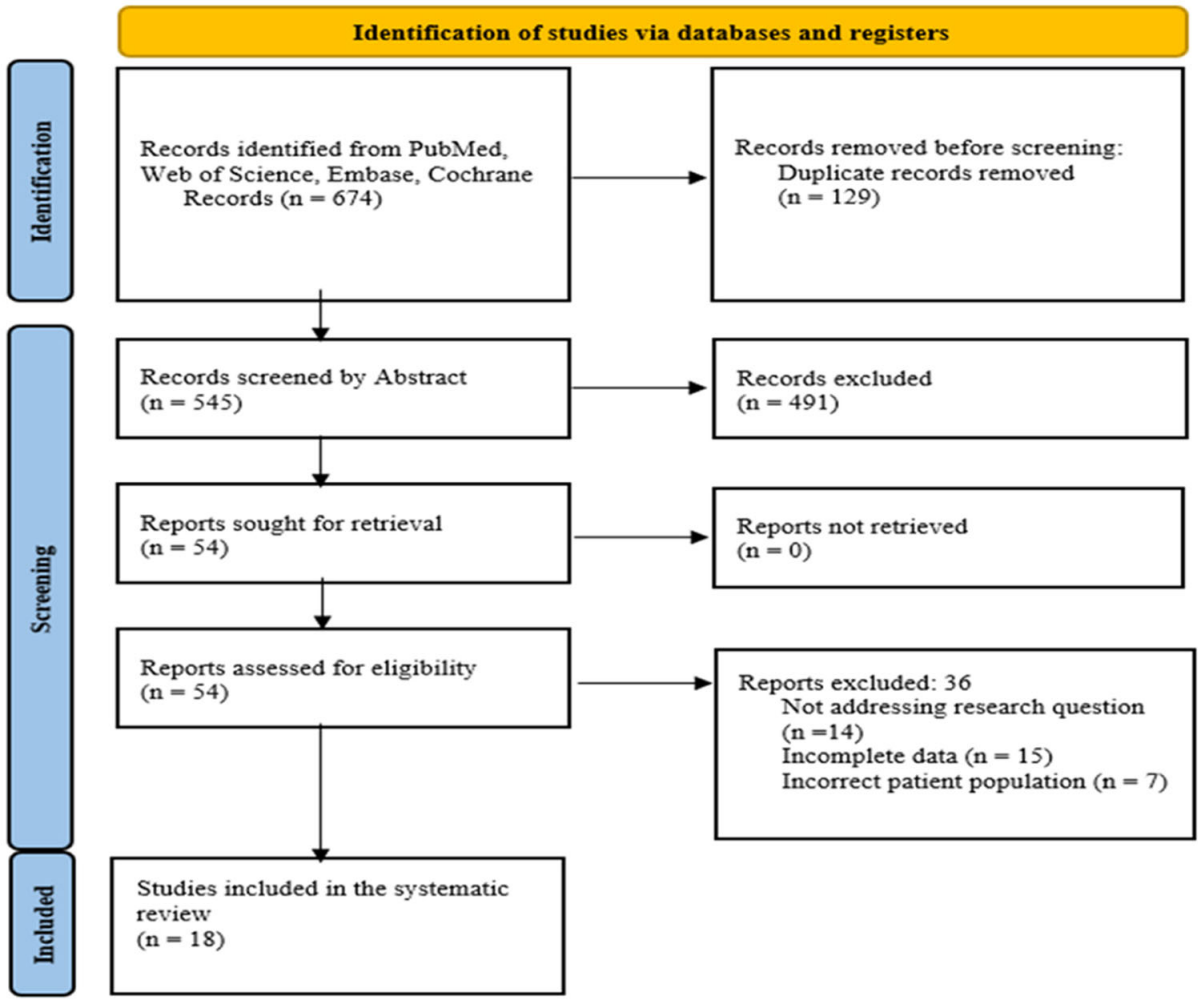
Flowchart of the search and screening process.

A total of 674 studies were retrieved after searching the various databases. Following the removal of duplicated articles (*n* = 129), 545 studies were further screened by title and abstract. Of these, 491 studies were omitted as they did not match the research question or were reviews, case reports, conference abstracts, letters to editors, or pediatric research. A total of 54 studies were selected, and their full texts were obtained. Finally, 18 articles were included in the final review and the analysis after being reviewed for PICO (Population, Intervention, Comparison, and Outcome) using the retrieved full‐text articles (Figure 1).

### 3.2 Study characteristics

Of the 18 articles, 6 articles were RCTs and 12 were observational studies. Several studies were conducted (*n* = 12) in multiple centers, whereas six studies were carried out at a single center (Table 1).

**Table 1:** Baseline characteristics of included studies.

Study ID	Study design	Follow‐up duration	Total sample size	No. of hours from first medical contact to Cath lab	Outcomes evaluated
Omer/2022/Egypt	RCT	30 days	200	Group 1 (*n* = 100): <2 h	All cause mortality
					Arrhythmia
				Group 2 (*n* = 100): >2 h	Heart failure
Jack/2021/USA	Observational: Pro Cohort	10 days	50	Group 1 (n = 20): <4 h	All cause mortality
					Arrhythmia
				Group 2 (*n* = 30): >6 h	Heart failure
Hauw‐Berlemont/2022/France	RCT	180 days	279	Group 1 (*n* = 141): <2 h	180‐day survival rate
					Arrhythmias
					Heart failure
					Shock
				Group 2 (*n* = 138): >2 h	Length of hospital stay
Irene/2017/The Netherlands	Observational: Retro Cohort	30 days	507	Group 1 (*n* = 291): <3 h	All cause mortality
				Group 2 (*n* = 216): >3 h	
Dankiewicz/2015/International (Europe, Australia)	Observational: Retro cohort	180 days	544	Group 1 (*n* = 292): <6 h	All cause mortality
				Group 2 (*n* = 252): >6 h	Neurological outcome
Lemkes/2019/The Netherlands	RCT	90 days	538	Group 1 (*n* = 273): <2 h	Survival rate
					Myocardial injury
					Acute kidney injury
				Group 2 (*n* = 265): >2 h	Neurological status
Jentzer/2018/United States	Observational: Pro cohort	Hospital discharge	599	Group 1 (*n* = 283): <24 h	Survival at hospital discharge
				Group 2 (*n* = 316): >24 h	
Kern/2020/United States	RCT	180 days	99	Group 1 (*n* = 49): <2 h	Survival to hospital discharge
				Group 2 (*n* = 50): >2 h	Composite adverse events
Kern/2015/United States	Observational: Retro Cohort	Hospital discharge	548	Group 1 (*n* = 183): <2 h	Survival to hospital discharge
				Group 2 (*n* = 365): >2 h	Functional status
Elfwe'n/2019/Sweden	RCT	24 h	78	Group 1 (*n* = 38): <24 h	All‐cause mortality at 24 h
				Group 2 (*n* = 40): >24 h	
Elfwe'n/2018/Sweden	Observational study: Pro Cohort	3 years	799	Group 1 (*n* = 275): <24 h	Survival at 30 days, 1 year and 3 years
				Group 2 (*n* = 524): >24 h	
Kleissner/2015/Czech Republic	Observational Study: Pro Cohort	180 days	99	Group 1 (*n* = 25): <2 h	Survival rate
				Group 2 (*n* = 74): >2 h	Neurological status
Aissaoui/2018/France	Observational: Retro Cohort	Hospital Discharge	1502	Group 1 (*n* = 1038): Age <65	Neurological status at discharge
				Group 2 (*n* = 281): Age 65–75	
				Group 3 (*n* = 183): Age >75	
Patel/2016/United States	Observational: Retro Cohort	Hospital Discharge	407974	Group 1 (*n* = 143,688): Underwent CAG	Use of coronary angiography and PCI
				Group 2 (*n* = 264,286): Did not have CAG	Survival at hospital discharge
Hollenbeck/2013/United States	Observational: Pro cohort	Hospital discharge	269	Group 1 (*n* = 122): Early CC <24 h	Survival rate
					Neurological outcome
				Group 2 (*n* = 147): Delayed CC >24 h	Heart failure
Desch/2021/Germany	RCT	30 days	530	Group 1 (*n* = 265): Immediate CAG	All cause mortality
				Group 2 (*n* = 265): Delayed CAG	Neurological status
Patterson/2017/United Kingdom	RCT	30 days	33	Group 1 (*n* = 18): < 24 h	All cause mortality
				Group 2 (*n* = 15): >24 h	Neurological status
Vadeboncoeur/2018/United States	Observational: Pro Cohort	Hospital Discharge	1230	Group 1 (*n* = 706): No CAG	Survival at discharge
				Group 2 (*n* = 157): CAG with PCI	Neurological status
				Group 3 (*n* = 367): CAG without PCI	
Bougouin/2017/France	Observational: Retro cohort	Hospital discharge	1410	Group 1 (*n* = 667): CAHP <150	Survival at discharge
				Group 2 (*n* = 469): CAHP 150–200	Neurological status
				Group 3 (*n* = 274): CAHP >200	
Kim/2018/South Korea	Observational	30 days	227	Group 1 (*n* = 112): CAG <2 h	Survival rate
				Group 2 (*n* = 115): CAG 2–24 h	Neurological status
Cronier/2011/France	Observational: Pro cohort	Hospital discharge	111	Group 1 (*n* = 50): STEMI	Survival rate
				Group 2 (*n* = 61): NSTEMI	Neurological status
Lim/2011/Australia	Observational: Retro cohort	12 months	5189	Group 1 (*n* = 88): OHCA	All cause mortality
					Renal failure
					Heart failure
				Group 2 (*n* = 5101): Non‐OHCA	Bleeding

Across all the studies, a total of 335,737 patients were included in the final analysis. The number of patients in each study varied from 33 to 325,563, with the median number of patients in a single study being 541 patients. The average age in each study varied from 57 to 71 years, with the median being 65. Males represented most of the patients in all studies. Some studies were more comprehensive than others in detailing the characteristics of the included participants (Table S1).

### 3.3 Outcomes

Meta‐analysis was performed using a random effect model, effect size was calculated for both primary and secondary outcomes in terms of risk ratios and log risk ratios. Heterogeneity in the analysis was assessed by calculating the τ^2^ and measuring the prediction interval from the statistics, moreover, I^2^ was also used as the measure of heterogeneity.

### 3.4 Primary outcome

**Figure 2:**
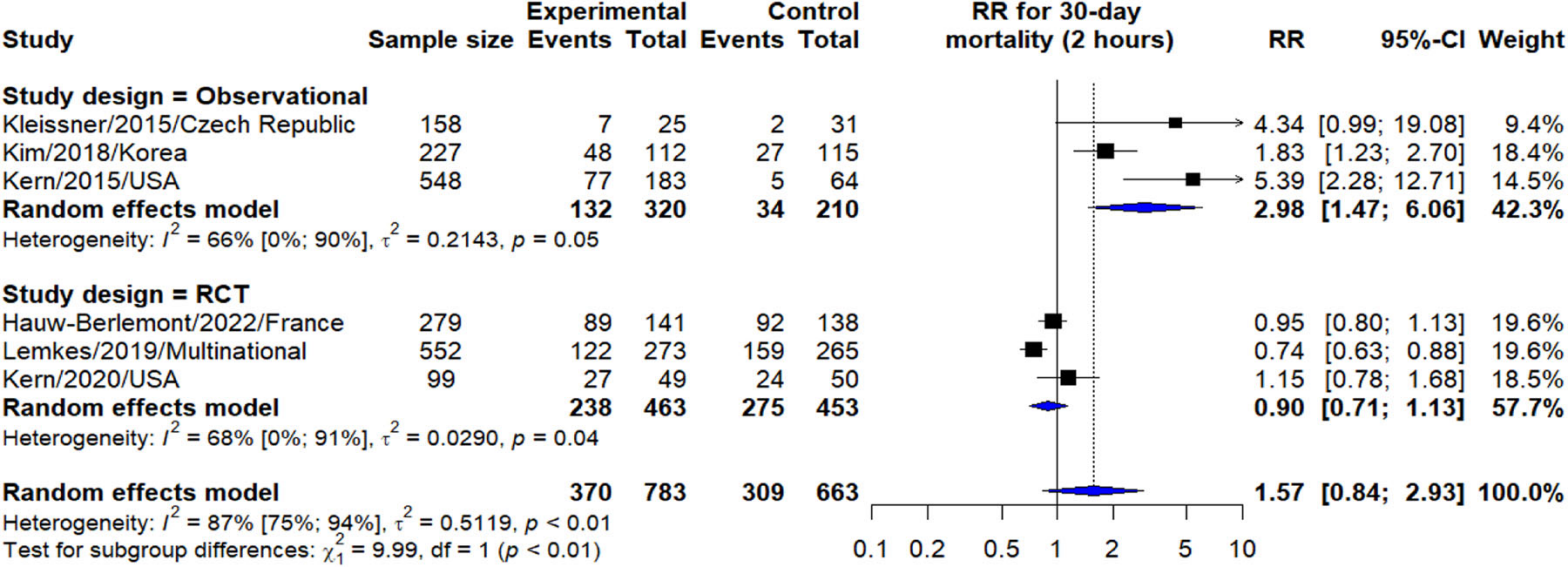
Early versus delayed CAG outcome on 30‐day mortality. CAG, coronary angiography.

In our meta‐analysis, we have found that those who underwent early CAG (≤2 h) had an increase in the 30‐day mortality, compared to the those who underwent delayed CAG (>2 h) (relative risk [RR]: 1.57, 95% CI: 0.84–2.93), yet not statistically significant. This effect was mainly derived from the findings of the observational studies (RR: 2.98, 95% CI: 1.47–6.06) as randomized trials have found equivocal effect between both groups in terms of 30‐day mortality (RR: 0.90, 95% CI: 0.71–1.13) (Figure 2).

**Figure 3:**
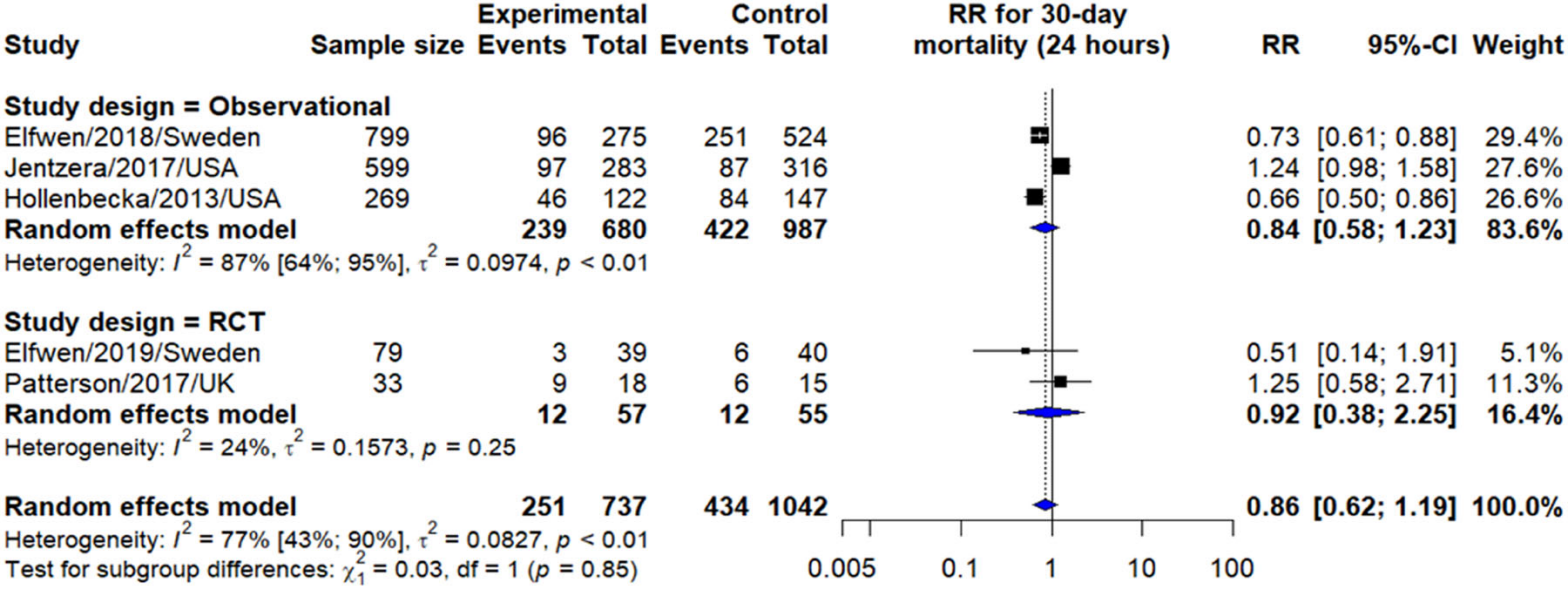
Early versus delayed CAG outcome on 30‐day mortality. CAG, coronary angiography.

On the other hand, when comparing those who underwent CAG within 24 h of OHCA to those who underwent CAG after 24 h, we observed a trivial reduction in risk of 30‐day mortality (RR: 0.86, 95% CI: 0.62–1.19), yet statistically insignificant. No significant was observed between findings from observational and randomized trials (Figure 3).

In two observational studies which used different cutoffs, we have found that: (1) in the study conducted by Irene et al., they defined the cutoff as 3 h from the OHCA and have found that those who underwent CAG within 3 h had lower mortality than others (RR: 0.72, 95% CI: 0.57–0.91) (Figure S1); (2) Dankiewicz et al.^27^ have defined early versus delayed CAG using a cutoff of 6 h and they observed lower 30‐day mortality for patients who underwent CAG within 6 h (RR: 0.39, 95% CI: 0.32–0.48) (Figure S1).

### 3.5 Secondary outcome

**Figure 4:**
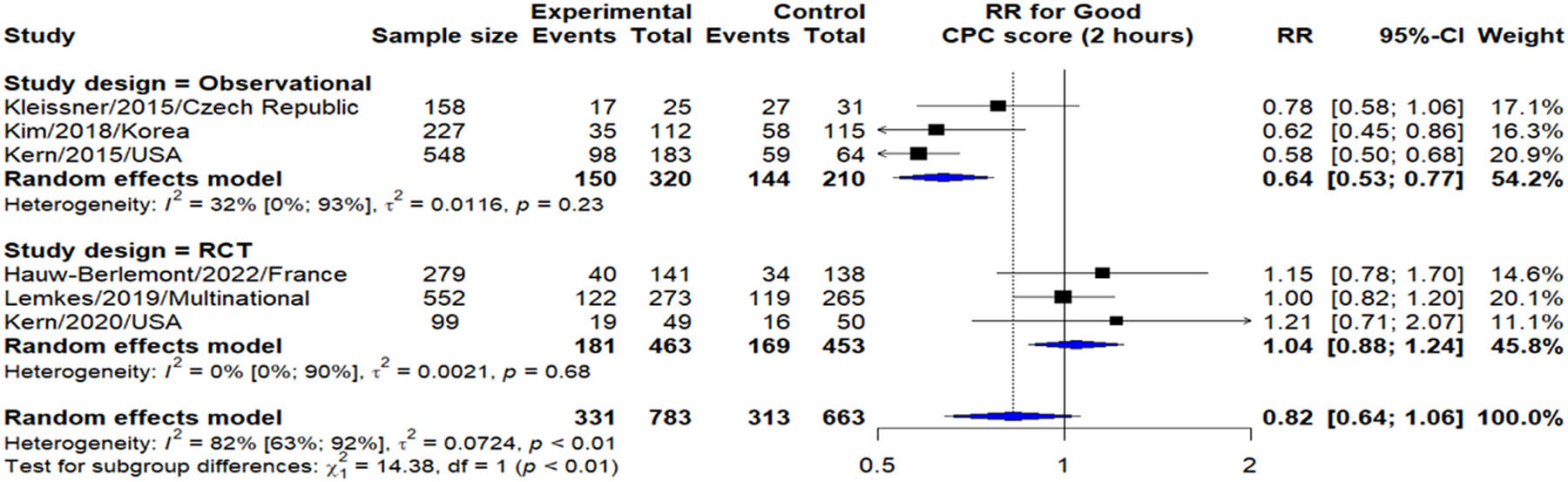
Early versus delayed CAG outcome on neurological outcomes. CAG, coronary angiography.

Compared to those who underwent CAG >2 h, we have found that undergoing CAG within 2 h of OHCA had insignificant reduction in probability of good CPC score (RR: 0.82, 95% CI: 0.64–1.06). Yet, it is to be noted that results from observational studies have demonstrated that performing CAG within 2 h of OHCA was significantly associated with lower risk of good CPC score (RR: 0.64, 95% CI: 0.53–0.77) (Figure 4).

**Figure 5:**
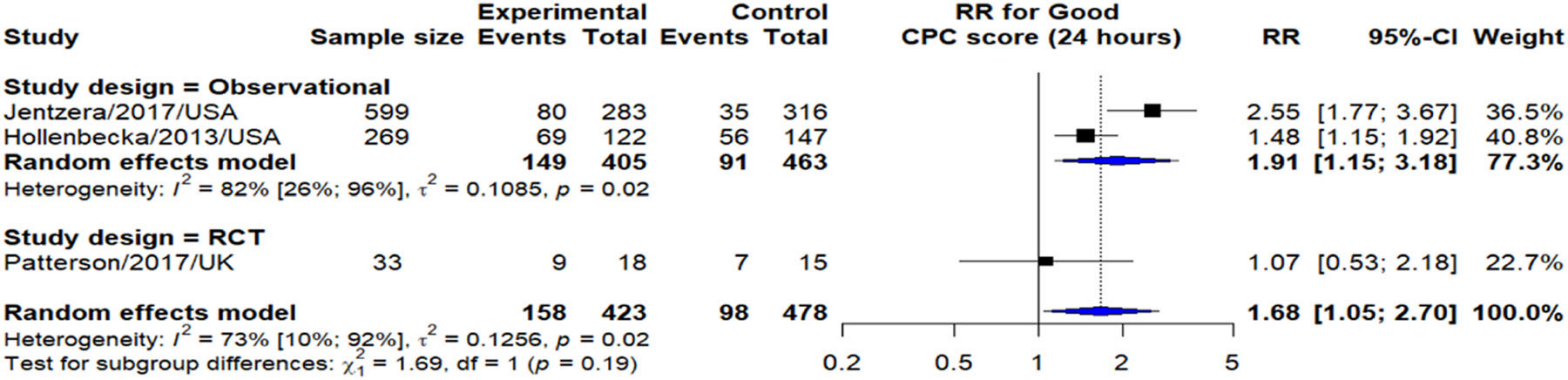
Early versus delayed CAG outcome on 30‐day mortality. CAG, coronary angiography.

Again, when using the cutoff of 24 h, we observed a significant increase in probability of good CPC score when CAG was performed within 24 h (RR: 1.68, 95% CI: 1.05–2.70). This effect was mostly because of the observational studies results (RR: 1.91, 95% CI: 1.15–3.18) (Figure 5).

## 4 DISCUSSION

The benefit of early CAG is still debatable in patients' achieving ROSC after OHCA without ST elevation on ECG. The reason for this debate is that there are various studies that favor early CAG, including RCTs.^28^, ^29^, ^30^, ^31^ Having said this, many studies have shown no benefit of urgent CAG in reducing mortality or improving neurological outcomes.

The AHA's 2015 updated recommendations state emergency CAG is reasonable for select adult patients with electrical or hemodynamic instability and who are comatose after an OHCA with no ST elevation (class IIa recommendations).^29^ According to the 2020 European Society of Cardiology (ESC) guidelines outlined by the task force for the management of acute coronary syndrome (ACS) in patients presenting without persistent ST‐elevation, if the post‐resuscitation ECG does not show ST‐elevation, patients should undergo urgent angiography within 2 h after a quick evaluation to exclude noncoronary cause. The guideline also recommends considering factors related to poor neurological outcomes when considering urgent CAG. This is because severe neurologic impairment may offset any benefit from immediate coronary reperfusion. The presence of an unwitnessed cardiac arrest, initial non‐shockable rhythm, prehospital team's late arrival without basic life support (>10 min) or more than 20 min of advanced life support without return to spontaneous circulation should all be taken strongly into account to argue against invasive coronary intervention.^32^

Our study has added discreet evidence to the already available literature, as we have included two recently reported RCTs on this topic in our final analysis (TOMAWAHAK and EMERGE).^8^, ^14^ Our results showed that there was no significant reduction in mortality when early CAG was performed in a subgroup of patients who had achieved ROSC and had no ST elevation after OHCA. Moreover, neurological recovery was similar in patients who underwent early or late CAG. These results are well in line with the multiple RCTs, including TOMAHAWK and EMERGE.^8^, ^14^ Preliminary results from other similar ongoing trials, DISCO (Direct or Subacute Coronary Angiography in Out‐of‐Hospital Cardiac Arrest) and ARREST (Randomized Trial of Expedited Transfer to a Cardiac Arrest Center for Non‐ST Elevation OHCA), also suggest that early CAG showed no benefit in short‐term survival rate when compared to delayed CAG.^16^, ^20^

Although our results are consistent with most studies included, there are some aspects that need to be considered in the included papers. For example, while the TOMAHAWK trial had a good randomization process, baseline characteristics were balanced, clearly defined and focused end point and was conducted in multiple centers across Europe increasing,^8^ the trial had several limitations such as the unblinding nature of the investigation and the higher rates of cross‐over from delayed/selective cohort to CAG within 24 h due to cardiogenic shock, large myocardial injury, new ST elevation, electrical instability, and violating study protocol. In addition, the CI for secondary outcomes was not adjusted for multiplicity leading to multiplicity bias, and as the authors rightly mentioned secondary outcomes are hypothesis‐generating only, limiting external applicability of the study.^12^ Similarly, the French multicentric EMERGE trial^14^ had some limitations mainly being underpowered due to the nonachievement of the pre‐planned sample size with difficulties obtaining informed consent and overestimation of cardiac arrests meeting the inclusion and exclusion criteria. Physicians not being blinded was another limitation of the study. Furthermore, the angiograms, echocardiograms, and follow‐up visits were not assessed by a core laboratory.^33^

This data supports the hypothesis that clinical factors other than early CAG may have an influence on survival in patients with non‐ST elevated OHCA, emphasizing the importance of ICU management and careful selection of patients for interventional revascularization strategies. Researchers hypothesize that the paucity of benefits with early CAG might be attributable to the absence of ACS. As evident from a study, coronary artery vasospasm was observed in only 11.7% of on‐ST elevated OHCA patients.^34^ Similarly, the COACT trial found that only 14.8% of the sample population had coronary lesions that met the criteria for ACS.^16^, ^35^ Hence, it is essential to focus patient selection on identifying individuals who would benefit most from early interventional revascularization techniques.

## 5 LIMITATIONS

The present study acknowledges certain limitations in the interpretation of its findings. Including observational studies, which are considered weaker evidence compared to randomized controlled trials, may restrict the generalizability and robustness of the results. Observational studies are prone to biases and confounding, making it challenging to establish causal relationships. However, the study aimed to provide a comprehensive overview by incorporating both RCTs and observational studies. Subgroup analyses were conducted to address this limitation, examining variables and potential confounders to gain a nuanced understanding of the associations. Random effects models were employed to account for heterogeneity across studies and enhance the reliability of the findings. Despite these adjustments, caution should be exercised when applying the results to broader populations or contexts. Further research using rigorous experimental designs and larger sample sizes is needed to strengthen the evidence base and improve generalizability. However, we sub‐grouped the analyses to account for this issue and used random effects models. Second, there was variation between the definitions reported in different studies. Few studies used the term “early CAG” to refer to operations conducted within 2 h of an accident, whereas others used 3, 6, or 24 h. To overcome this issue, we performed three analyses for different definitions as follows: 2 h, 24 h, and all definitions other than 2 h. Third, various trials studied the 30‐, 90‐, and 180‐day mortality rates that might limit the generalizability of a single‐window period. Aside from that, we only evaluated the mortality and neurological status of the patients. Other factors such as procedure‐associated complications, time of hospital stay, and quality of life, were not assessed due to the paucity of the data. Finally, a previous systematic review and meta‐analysis addressed the same research questions as ours^36^; however, they have not included observational studies, which may add another perspective to the problem.

## 6 CONCLUSIONS

After analyzing the relevant literature, we conclude that performing emergency CAG fails to reduce mortality and improve neurological outcomes in patients with OHCA without ST elevations on post‐ROSC ECG. Therefore, in this cohort of patients, early CAG should not be a preferred approach while evaluating and managing the cause of OHCA, and nonemergent delayed CAG should be performed to look for a cardiac cause of OHCA.

## AUTHOR CONTRIBUTIONS

**Khalid Rashid**: conceptualization; investigation; project administration; supervision; writing—original draft. **Muhammad Aamir Waheed**: data curation; formal analysis; writing—original draft; writing—review & editing. **Farrukh Ansar**: data curation; investigation; methodology; resources; software; visualization; writing—original draft; writing—review & editing. **Abdelrahman M Makram**: formal analysis; software; visualization. **Ahmedyar Hasan**: investigation; methodology; writing—review & editing. **Shahab Ahmed**: project administration; validation; writing—review & editing. **Saad Tariq Khan**: investigation; methodology; writing—review & editing. **Aamer Ubaid**: investigation; resources; visualization. **Ahmad Al Ibad**: investigation; writing—review & editing. **Rabia Basri**: methodology; validation; writing—review & editing. **Omar Mohamed Makram**: formal analysis; writing—original draft. **Yahya Khan**: investigation; validation; writing—review & editing. **Nabhan Rashad**: data curation; investigation; project administration. **Abdelnaser Elzouki**: project administration; supervision; validation.

## CONFLICT OF INTEREST STATEMENT

The authors declare no conflicts of interest.

## ETHICS STATEMENT

This is a systematic review and does not require an ethical statement. However, as per guidelines, this project was registered with PROSPERO Ref No. = CRD42022327833.

## TRANSPARENCY STATEMENT

The lead author Khalid Rashid affirms that this manuscript is an honest, accurate, and transparent account of the study being reported; that no important aspects of the study have been omitted; and that any discrepancies from the study as planned (and, if relevant, registered) have been explained.

## Supporting information

Supporting information.

## Data Availability

The data can be uploaded to any repository as advised by Editor.
