# Supplementary material for: Early coronary angioplasty fails to lower all‐cause mortality in patients with out‐of‐hospital cardiac arrest without ST‐segment elevation: A systematic review and meta‐analysis
Source: Health Sci Rep. 2024 Jan 30;7(2):e1379. doi: 10.1002/hsr2.1379 (PMC10828130; doi:10.1002/hsr2.1379)
Supplement: Supplementary file 1 — Supporting information. [file HSR2-7-e1379-s001.docx]

**Article ID: HSR21379**

**Article DOI: hsr2.1379**

**Figure S1: Early vs Delayed CAG Outcome on 30-day mortality**


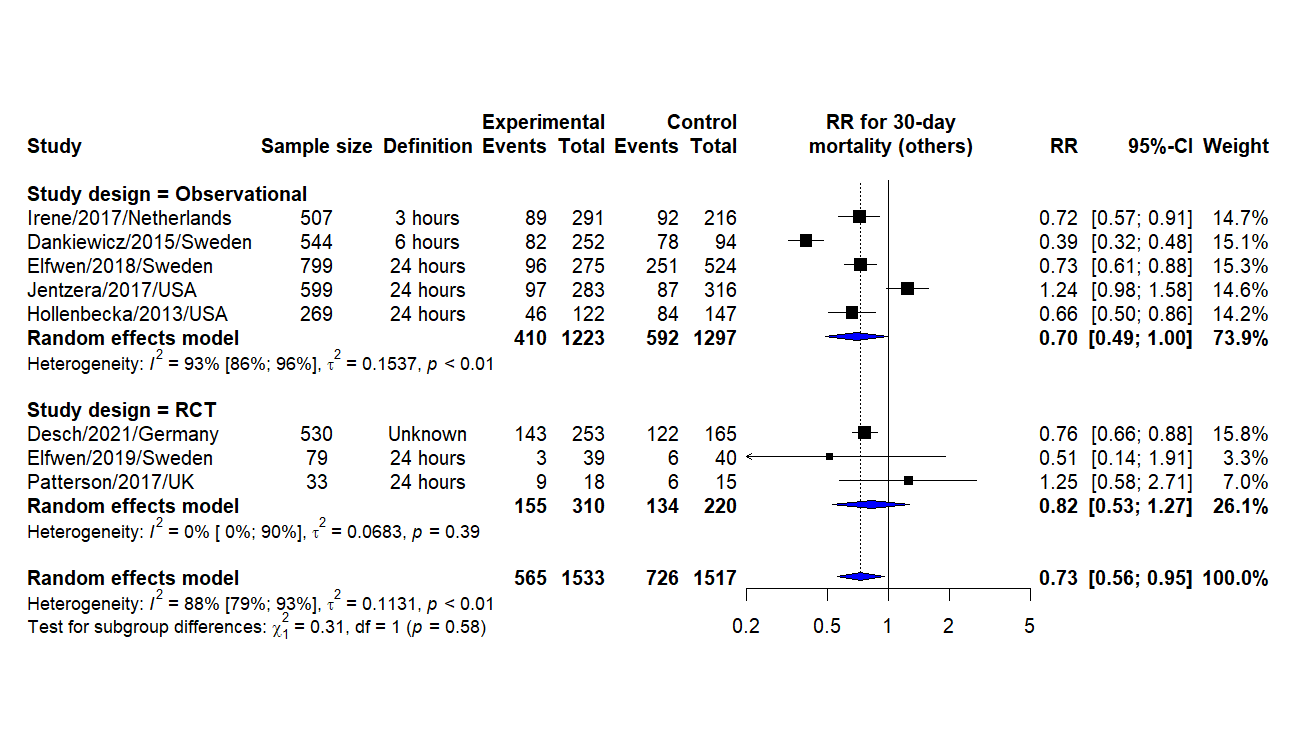


**
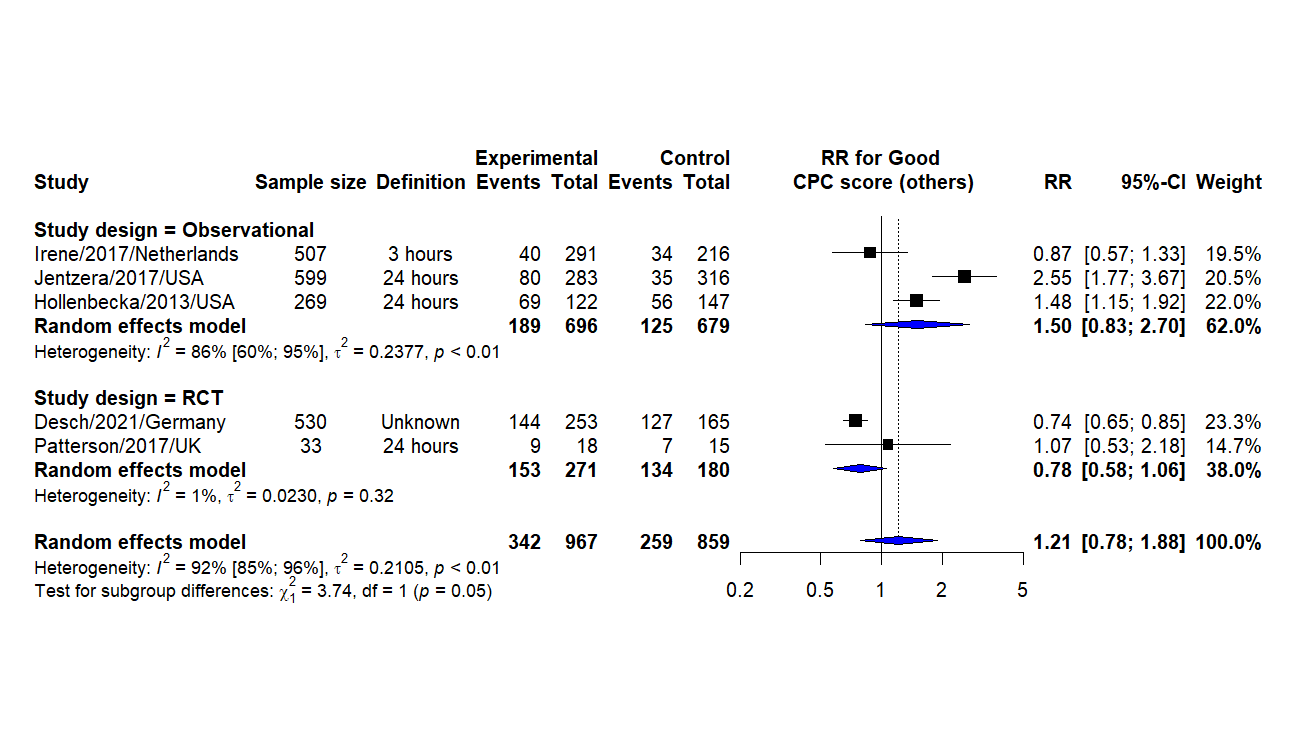
Figure S2: Early vs Delayed CAG Outcome on neurological outcomes**

**Table S1: Patient Characteristics of included studies**

| **First Author of Study** | **Number of patients** | **Average age** | **Males** | **HTN** | **Diabetes** | **Stroke** | **CHF** | **CABG** | **PCI** | **CAD** | **STEMI** | **NSTEMI** | **Shockable rhythm (VT/VF)** | **Asystole/PEA** | **Bystander CPR** |
| --- | --- | --- | --- | --- | --- | --- | --- | --- | --- | --- | --- | --- | --- | --- | --- |
| J.S. Lemkes et al., 2019 | Cohort: 522 (Early CAG: 273) | 65 | 425 (81%) | 257 (49%) | 99 (19%) | 34 (7%) | 57 (11%) | 67 (13%) | 106 (20%) | 195 (37%) | N/A | N/A | N/A | N/A | N/A |
| Nadia Aissaouia et al., 2018 | Cohort: 1502 | 65 | 1200 (80%) | N/A | N/A | N/A | N/A | N/A | 1168(78%) | 802 (53%) | 856 (57%) | 646 (43%) | N/A | N/A | 1055(70%) |
| Tyler F. et al., 2018 | Cohort: 1881 | 63 | 1289 (69%) | N/A | N/A | N/A | N/A | N/A | N/A | N/A | N/A | N/A | 930 (49%) | 951 (51%) | 677 (36%) |
| Youn-Jung Kim et al., 2018 | Cohort: 227 | 57 | 170 (75% | 89 (39%) | 54 (24%) | N/A | 11 (5%) | N/A | 49 (22%) | 24 (11%) | N/A | N/A | 104 (49%) | 123(54%) | 115(51%) |
| Jacob C. Objentzera et al., 2017 | Cohort: 599 | N/A | N/A | N/A | N/A | N/A | N/A | N/A | 151(25%) | N/A | N/A | N/A | N/A | N/A | N/A |
| Wulfran Bougouin et al., 2017 | Cohort: 1410 | 62 | 972 (69%) | N/A | N/A | N/A | N/A | N/A | N/A | 422 (30%) | N/A | N/A | 712 (50%) | 698(49%) | 907 (64%) |
| Isabelle Irene et al., 2017 | Cohort: 507 | 63 | 399 (79%) | 168 (33%) | 90 (18%) | 31 (6%) | N/A | 39 (8%) | 61(12%) | N/A | N/A | N/A | 275 (54%) | 232(46%) | N/A |
| S. Desch et al., 2021 | Cohort: 530 | 70 | 369 (68%) | 323 (61%) | 145 (27%) | N/A | N/A | 44(PREV CABG) | 98 (Prev PCI) | N/A | N/A | 530 (100% | 268 (51%) | N/A | 294 (55%) |
| Karl B. Kern et al., 2019 | Cohort: 99 | 65 | 78 (14.7% | 55 (10%) | 27 (5%) | N/A | 14 (3%) | 6 (1%) | 24 (5%) | 32 (6%) | N/A | 99 (19%) | 75 (14%) | 8 (2%) | 70(13% |
| Nish Patel et al., 2016 | Cohort: 325,563 | 66 | 196,965 | 158,875 | 91,483 | N/A | 148457 | N/A | N/A | N/A | N/A | N/A | 325563 | 0 | N/A |
| Ludvig Elfwe´n et al., 2019 | Cohort: 79 | 71 | 53(67%) | N/A | 16(20%) | 10 (12%) | 13 (16%) | 6 (7%) | 11 (15.4%) | N/A | N/A | 71 (100%) | 22 (31%) | 12 (17%) | 58 (82%) |
| Ludvig Elfwe´n et al., 2018 | Cohort:799 | 66 | 629 (79%) | 206 (26%) | 112 (14% | 49 (6%) | 147 (18%) | N/A | 25 (3%) | N/A | N/A | 799 (100%) | N/A | N/A | 578 (72%) |
| Tiffany Patterson et al., 2017 | Cohort: 33 | N/A | N/A | N/A | N/A | N/A | N/A | N/A | N/A | N/A | N/A | 33 (100%) | N/A | N/A | N/A |
| Martin Kleissner et al., 2015 | Cohort: 158 (59 excluded stemi/lbbb) | 59 | 133 (84%) | N/A | N/A | N/A | N/A | N/A | N/A | 58 (37%) | 59 (37%) | 36 (23%) | NA | 34 (22%) | 75 (47%) |
| Karl B. Kern et al., 2015 | Cohort: 548 (NSTEMI) | 61 | 512 (93%) | N/A | N/A | N/A | N/A | N/A | 209 (38%) | N/A | N/A | N/A | NA | 99 (18%) | 229 (48%) |
| J. Dankiewicz et al., 2015 | Cohort: 544 | 66 | 431 (97%) | 228 (51%) | 91 (20%) | 44 (10%) | 47 (11%) | N/A | N/A | N/A | N/A | N/A | 409 (75%) | 135 (24%) | 384 (62%) |
| Ryan D.Hollenbecka et al., 2013 | Cohort: 269 | 60 | 191 (71%) | N/A | N/A | N/A | N/A | N/A | N/A | N/A | 156 (58%) | N/A | 269 (100%) | NA | 152 (57%) |
| Caroline Hauw-Berlemont et al., 2022 | Cohort: 279 | 65 | 195 (70%) | N/A | N/A | N/A | N/A | N/A | N/A | N/A | N/A | N/A | NA | 183(66%) | 191 (71%) |
